# A standard procedure for constructing a multi-level social vulnerability index using CLSA and SOS data as working examples

**DOI:** 10.1371/journal.pone.0315474

**Published:** 2024-12-13

**Authors:** Jasmine C. Mah, Olga Theou, Mario Ulises Perez-Zepeda, Jodie L. Penwarden, Judith Godin, Kenneth Rockwood, Melissa K. Andrew

**Affiliations:** 1 Department of Medicine, Dalhousie University, Halifax, Nova Scotia, Canada; 2 Division of Geriatric Medicine, Dalhousie University, Halifax, Nova Scotia, Canada; 3 Geriatric Medicine Research, Dalhousie University and Nova Scotia Health, Halifax, Nova Scotia, Canada; 4 School of Physiotherapy, Dalhousie University, Halifax, Nova Scotia, Canada; 5 Research Department at Instituto Nacional de Geriatría, Ciudad de México, México; University of Naples Federico II, ITALY

## Abstract

**Background:**

The construct of *social vulnerability* attempts to understand social circumstances not merely as a descriptor, but as a predictor of adverse health events. It can be measured by aggregating social deficits in a social vulnerability index (SVI). We describe a standard procedure for constructing a multi-level SVI using two working examples.

**Methods:**

First, we describe a six-step approach to constructing a SVI. Then, we conducted a secondary analysis of a clinical dataset (Canadian Immunization Research Network’s Serious Outcomes Surveillance Network (SOS)) and a population-based dataset (Canadian Longitudinal Study on Aging (CLSA)). In both datasets, we construct SVIs, use descriptive statistics to report distributions by age and sex, and perform a multivariable linear regression of social vulnerability on frailty.

**Results:**

Procedures for drafting a list of candidate social items, selecting deficits for inclusion, and screening deficits to meet inclusion criteria were applied to yield a 18-deficit SVI for the SOS and 74-deficit SVI for the CLSA. Deficits in each SVI were re-scored between 0 and 1, where 1 indicates the greater risk. Finally, the sum of all deficits is calculated into an index. In the SOS, SVI was associated with age only for females and was weakly associated with frailty (r = 0.26, p<0.001). In the CLSA, SVI was associated with age for both sexes and moderately associated with frailty (r = 0.41, p<0.001).

**Conclusion:**

We present a standard method of constructing a SVI by incorporating factors from multiple social domains and levels in a social-ecological model. This SVI can be used to improve our understanding of social vulnerability and its impacts on the health of communities and individuals.

## Background

The conditions in which people are born, live, work and age collectively influence their ability to anticipate, cope, resist and recover from an adverse event [1]. *Social vulnerability* provides a way of understanding the social environment not merely as a descriptor, but as an attempt to quantify an individual’s or community’s relative vulnerability to changes in their environment, social circumstances, health, or functional status [2]. In short, when all other non-social factors are equal, how do disadvantageous social circumstances lead to a community being disproportionally devastated by an epidemic or to an individual being unable to recover in the expected timeframe following an adverse health event?

Social circumstances are complex; there are many social factors, multiple layers of social factors from personal supports to neighbourhood dynamics, and numerous potentially unforeseen interactions among such factors. While measuring social vulnerability may be perceived as challenging, conveniently, social vulnerability can readily be measured using available data. One way to estimate social vulnerability is through an index aggregating social factors. An index approach has several benefits. It can provide a holistic picture of social circumstances by including different categories of social factors (e.g., socioeconomic status, social engagement, social capital), by avoiding arbitrarily separating related factors into distinct categories and by accounting for gradations in social vulnerability [2, 3].

Social vulnerability indices (SVIs) are used to measure complex social circumstances associated with health outcomes. The SVI employed by the Agency for Toxic Substances and Disease Registry / Centers of Disease Control and Prevention [4] is widely used, and in the United States, associated with many adverse outcomes, such as in SARS-Cov-2 [5], surgery [6], and heart failure re-admissions [7]. Another well-known SVI by Cutter and colleagues [8] has been adapted and shown to be associated with cancer risk [9] and Lyme disease incidence [10]. However, neither of these routinely used social vulnerability tools were initially developed for use in health or medical fields. Further, a recent scoping review suggested a SVI might be strengthened if composed of social factors which reflect vulnerabilities at the individual, household, and community levels [11]. To address these gaps, we aim to describe a standard procedure for constructing a multi-level SVI with relevance to the health of individuals using a social deficit approach. We provide two working examples of constructed SVIs using this approach.

## Methods

### Theory

Our operationalization of social vulnerability builds upon social capital theory, especially that such capital can be deployed in times of need. Broadly, social capital is the organization of social structures and how these structures facilitate actions of stakeholders in the society [12]. Social capital is a collective resource; like economic capital, harnessing social capital brings advantages for the wealthy or powerful through their entrenched networks and institutionalized relationships [13]. It consists in social support, social engagement and access to resources (including economic capital) [14]. Adequate social capital is productive; in its absence, achievement of desired ends would not be possible [12]. To illustrate, following a hip fracture, economic capital to purchase a wheelchair or renovate a home with a ramp improves function, but so will social capital in the form of free exchanges of food, time or company provided by friends, family and community members. Whether social capital exists at the level of the individual or the collective has been the subject of much debate. We see social capital as related both to characteristics of the individual (e.g., educational), and also to other aspects at the level of culture and the environment (e.g., neighbourhood safety). These related but distinct non-medical factors—the social determinants of health [15]—exist with social capital on a continuum from individual to collective [14].

A socio-ecological framework [16], wherein an individual is nested within expanding spheres of social influence, offers a useful way to think about how multiple social factors influence health [2]. Social factors exist on a continuum of multiple levels of social influence—from the individual to family and friends, neighbourhoods, and communities, and society at large—and contribute to overall social vulnerability [2]. For an adult seeking healthcare, at the *micro* level, social factors are their own health behaviours and their closest links with family and caregivers. We define the *meso* level according to Newman and Newman as the interrelations among two or more microsystems that then impact the individual [17]. Examples includes family-friends interactions, or friends-healthcare interactions (e.g., the health literacy of friends and relations and their access to resources and supports). The *exo* level refers to the available community supports available such as home care services or access to rehabilitation programs. The *macro* level encompasses the attitudes towards older adults in broader policy reflected in pension plans or universal health care [16]. A measure of social vulnerability must account for this complexity and include social factors across the continuum from an individual to a group level. Similarly, a lone marker of social circumstances cannot adequately reflect the multifaceted interactions between social factors; therefore, a global index of social vulnerability must incorporate multiple social determinants of health.

### Constructing the SVI

We describe six steps, and recommendations and considerations, in the construction of a SVI.

#### 1. Draft a list of candidate items for the SVI

*Recommendations*. Choose social factors, for potential inclusion as items in the index, with high face validity and comprehensibility supported by a good theoretical base, with evidence of the potential to adversely impact health outcomes. Lists of candidate social deficits may be procured based on existing data availability [18], but have also been generated through consensus with experts [19].

*Considerations*. For controversial social factors (e.g., retirement or rurality where the deficit state may be beneficial to some and detrimental to others), consult experts who are familiar with the population of interest.

#### 2. Select deficits for inclusion in the SVI

*Recommendations*. Items selected will become deficits in the index. Collectively, they must include a range of factors across multiple social domains and across multiple levels of social influence representing a holistic view of a person’s social circumstances.

- Include social deficits across multiple social domains. Examples of social domains are: access to material resources (e.g. income or socioeconomic status), social support (e.g. links to family, friends or community) and social engagement (e.g. participation in collective society) [14]. Social domains can also include examples from the social determinants of health such as income & social status, employment & working conditions, education & literature, childhood experiences, physical environments, social supports & coping skills, and access to health services [15]. Social domains can also be chosen by including previously reported items in measurement tools such as a social support index [20] or social isolation index [21], among others.
- Include social factors from multiple spheres or levels of influence across the continuum from individual to group levels [22]. A recent scoping review summarizing the composition of items in SVIs noted more than half of all SVIs included items reflective of individual socioeconomic status, but also prevalence of at-risk populations in a geographic region [11].
- Include factors that are both objective (e.g. living alone, number of close friends) and subjective (e.g. loneliness, availability of emotional support).

*Considerations*. Selecting deficits for inclusion in the SVI necessitates a balance between creating a robust measure (generally optimized with a greater number of items) and data availability (in the case of secondary analysis of existing datasets) or participant burden (in the case of prospective data collection). Some SVIs use personality factors (e.g., neuroticism) and lifestyle factors (e.g., exercise and diet); we see these as independent aspects of wellbeing and representing another dimension of health, not to be used in a SVI.

Choosing deficits based on statistical correlation to one another is not recommended. Deficits are not required to be correlated with one another. Items in the SVI are more appropriately considered causal variables rather than indicator variables [23]. For example, education and feelings of loneliness may not be correlated, but they both contribute to a person’s social vulnerability. Both the causal direction and the lack of correlation between the deficits renders factor analysis inappropriate. The selection of deficits rests on their potential contribution to social vulnerability rather than their intercorrelations.

#### 3. Code deficits for the SVI

*Recommendations*. The coding for each item in the SVI depends on its scale of measurement. Regardless, all social deficits receive a score from 0 to 1. A value of 1 indicates the greatest state of relative vulnerability to damage (e.g., education: never completed high school = 1 and completed high school or greater = 0). For intermediate responses, deficits may take a value of 0.5 (e.g. never completed high school = 1, completed high school only = 0.5, post-secondary education = 0). Ordinal items may rank into a score according to number of levels. For example, a deficit with four levels (e.g., never completed high school, completed high school, college or university, post-graduate education) would be coded 0 for the social factor characteristic that is most protective, 0.33 and 0.66 for middle states of vulnerability and 1 for the most detrimental characteristic for vulnerability. Continuous items may be categorized according to pre-established cut points or coded into a continuous score between 0 and 1. In our education example, if measured in years, >20 years of school would be coded 0 and decreasing number of years allocated a score according to the equation 1—(# years of school / 20 max years of school).

*Considerations*. The theoretical basis of coding deficits for the SVI combines a deficit accumulation principle [24] with social capital underpinnings. Living alone, for example, is not always considered a deficit (i.e., an adverse social circumstance increasing the risk of damage or prolonging recovery time following an adverse health event) and individuals can be content living alone. However, social capital refers to resources one can draw upon should a crisis occur. Through this lens, living alone does confer an increased risk of social vulnerability and can be coded as the highest deficit state.

Another challenge with social deficit coding is the lack of self-evident cut points. We suggest two options. First, cut offs for vulnerable states could depend on expert consensus drawing on literature and experience. Second, cut offs can be determined by mapping distribution of the social variable using basic statistical techniques. Individuals with the deficit beyond the 75^th^ percentile of a specific dataset could be coded as having the most vulnerable state for that variable. This latter is for datasets that are truly representative of a population. The coding represented here is similar to previous work [24, 25].

#### 4. Screen deficits that meet inclusion criteria

*Recommendation*. Screen the chosen social deficits for missingness. The threshold for missing values is contingent on the total number of social deficits available for the construction of the index, although >5% missing data is generally acceptable as a cut off to exclude the deficits in similar indices [26]. Allowing for high levels of missingness at the item level may result in losing observations when calculating the SVI scores, assuming no other methods of dealing with missing data is employed such as multiple imputation [27].

Screen deficits for prevalence. A rare social deficit in the population (i.e., <1%) could be combined with another deficit to avoid exclusion in the final calculations or inflating the denominator.

#### 5. Calculate the final SVI

*Recommendations*. The overall SVI score per individual is calculated by summing the coded values for all social items (reflection of the deficits) and dividing by the absolute count of items included in the SVI. Therefore, the final SVI also takes a value between 0 and 1, allowing for standardization and development of a common language as it pertains to social vulnerability.

*Considerations*. Calculate a final SVI only for individuals with sufficient data. Calculating a final SVI for individuals missing more than 20% of SVI items may not accurately reflect their social circumstances and may underestimate true social vulnerability. Using statistical methods of dealing with missing data such as multiple imputation can be considered [27].

Similar to the frailty index, our SVI builds in natural weighting. For example, individuals who are not married are more likely to score social deficits for living alone and having less social support. While there are benefits of weighting items in a SVI (e.g., gaining performance or separability measurement), we aim for the SVI to be highly generalizable across contexts.

#### 6. Report the SVI

*Recommendations*. Report the number of items and domains. List the deficits and their coding. Report distribution of the SVI. Report correlation with age and sex/gender. If a SVI is to be used in the same dataset or across multiple time series, it should consist of the same variables from one iteration to the next.

## Worked examples

### Samples

To demonstrate the standard approach described above, we calculate and compare SVIs in two separate datasets.

The first is a clinical dataset with a minimal number of social variables. The Canadian Immunization Research Network’s Serious Outcomes Surveillance (SOS) Network is a prospective dataset of Canadians hospitalized with acute respiratory illness in six Canadian provinces. Within the SOS dataset, we selected all individuals over the age of 65 years old admitted to hospital during the 2011–2012 influenza season. Ethics approval for the use of SOS data was obtained from the Capital Health Research Ethics Board (CDHA-RS/2010-123).

The second dataset is a weighted population-based dataset: the Canadian Longitudinal Study on Aging, a national, stratified, prospective study of over 50,000 community-dwelling Canadian women and men aged 45 to 85 years old at time of recruitment [28]. We draw on the complete sample of the CLSA using both the comprehensive and tracking cohort from the baseline CLSA assessment in 2011. This research has been conducted using the CLSA dataset Baseline Tracking Dataset version 3.3, the Baseline Comprehensive Dataset version 3.2 and CLSA Sample Weights Version 1.2. We classify each item in the SVI by the domains included in the CLSA (i.e., socio-demographic, home ownership, education, social networks, social support availability, social participation, income, built environment and psychosocial). For performing a secondary analysis of data, a waiver of ethics application was approved by the Nova Scotia Health Authority Research Ethics Board. Data was obtained from the CLSA agreement #1906015.

#### Statistical methods

We use descriptive statistics to report SVI distributions by age and sex and report Pearson correlation coefficients. We performed a multivariable linear regression of social vulnerability on frailty. Frailty is measured using frailty indices (FI) from previously published papers [29, 30]. We calculated Receiver Operating Characteristics (ROC) Curves [31] and area under the ROC curves (AUC) for SVI with frailty (using a FI cut off of 0.21) and mortality [32].

### Constructing the SVIs

**Fig 1:**
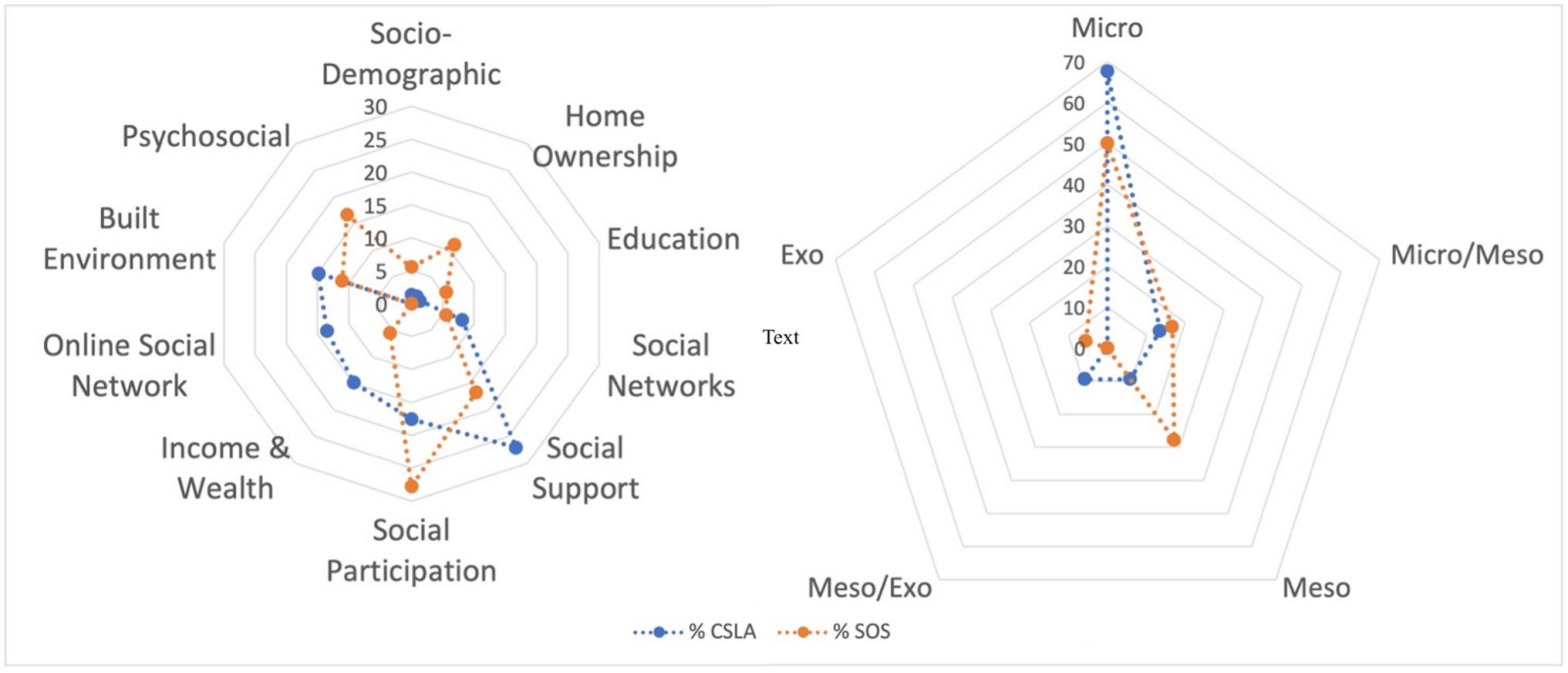
Proportion of items in the SOS and CLSA SVIs per social domain and per socio-ecological level.

The SVI calculated in the SOS Network dataset is an index composed of 18 items. Nine domains are represented by these 18 deficits when the CLSA social domain classifications are applied as shown in Table 1 and Fig 1. Of the 18 deficits, 13 were coded as dichotomous and the remainder were ordinal. The SOS SVI is an example of an index constructed based on data availability (see step 2 considerations).

**Table 1:** List of social items included in the serious outcomes surveillance network SVI.

	Item	Coding	Level
Socio-Demographic			
1	Current marital status	0 = married or common in law; 1 = single, divorced or widowed	Micro
Home Ownership			
2	Ever homeless	0 = no; 1 = yes	Micro
3	Lives in a rooming house, group home, shelter or is currently homeless	0 = no; 1 = yes	Meso
Education			
4	Highest level of education	0 = college, university bachelor, graduate, or professional degree; 0.33 = trades or apprenticeship; 0.67 = high school; 1 = less than high school	Micro
Social Networks			
5	Living alone	0 = no; 1 = yes	Micro
Social Support Availability			
6	Does the patient have someone to count on for help or support	0 = yes; 1 = no	Micro
7	Does the patient feel they need more help or support	0 = no; 1 = yes	Micro
8	Does the patient have someone to confide in	0 = yes; 1 = no	Micro
Social Participation			
9	How often patient participated in activities, groups or clubs in the community	0 = often (weekly); 0.5 = sometimes; 1 = never	Meso
10	Does the patient volunteer in the community	0 = yes; 1 = no	Meso
11	How often patient attends religious services	0 = often (weekly); 0.5 = sometimes; 1 = never	Meso
12	How often does the patient get together and socialize with friends	0 = often (weekly); 0.5 = sometimes; 1 = never	Micro / Meso
13	How often does the patient get together and socialize with family/relatives	0 = often (weekly); 0.5 = sometimes; 1 = never	Micro / Meso
Income			
14	Feels that income currently satisfies needs	0 = no; 1 = yes	Micro
Built Environment			
15	Does the patient say that most people can be trusted	0 = yes; 1 = no	Meso
16	Does the patient feel safe in their neighbourhood	0 = yes; 1 = no	Exo
Psychosocial			
17	Does the patient feel they have control over things that happen to them	0 = yes; 1 = no	Micro/ Meso
18	Does the patient feel lonely	0 = no; 1 = yes	Micro

The population-based SVI is constructed from 74 items that met criteria in the CLSA. The CLSA SVI is an index constructed based on the theories proposed in Steps 1, 2 and 3 given the abundance of social items for in the dataset. Initially, 84 items were drafted from the CLSA. Seven items did not meet screening criteria due to poor face validity (e.g. personality or lifestyle factors rather than social factors) or because the author group could not come to consensus on the item’s deficit state (e.g. retirement) The CLSA SVI is composed of deficits from ten social domains that were already established by the CLSA data collection process; deficits from the social support domain were the most prevalent (20/74) as seen in Table 2 and shown in Fig 1. Fig 1 also shows how each item relates to the socio-ecological framework and each level of social influence. Most deficits were coded as ordinal variables (55/74) and the remaining were coded as dichotomous. Screening of CLSA deficits (Step 4) for prevalence and missingness was possible due to large numbers and is available in S1 File. Other recreational activities, church activities and club and fraternal services were combined due to low individual prevalence. No items were excluded due to missing data due to the completeness of the CLSA dataset. Finally, one item was removed (i.e., social inequity) because it was not available in all waves of the CLSA and a SVI that can be used longitudinally should have the same items (Step 6).

**Table 2:** List of social items included in the Canadian Longitudinal Study on Aging SVI.

	Item	Coding	Level
Socio-Demographic			
1	Marital status	0 = yes; 1 = no	Micro
Home Ownership			
2	Home owner	0 = yes; 1 = no	Micro
Education			
3	Education	0 = college, university bachelor, graduate, or professional degree; 0.33 = trades or apprenticeship; 0.67 = high school; 1 = less than high school	Micro
Social Networks			
4	Living alone	0 = no; 1 = yes	Micro
5	Child contact frequency	0 = within the last day or two or all children live in household; 0.2 = within the last week or two; 0.4 = within the past month; 0.6 = within the past 6 months; 0.8 = within the past year; 1 = more than one year ago or no children	Micro
6	Siblings contact frequency	0 = within the last day or two or all siblings live in household; 0.2 = within the last week or two; 0.4 = within the past month; 0.6 = within the past 6 months; 0.8 = within the past year; 1 = more than one year ago or no siblings	Micro
7	Relatives contact frequency	0 = within the last day or two or all relatives live in household; 0.2 = within the last week or two; 0.4 = within the past month; 0.6 = within the past 6 months; 0.8 = within the past year; 1 = more than one year ago or no relatives	Micro
8	Friends contact frequency	0 = within the last day or two or all friends live in household; 0.2 = within the last week or two; 0.4 = within the past month; 0.6 = within the past 6 months; 0.8 = within the past year; 1 = more than one year ago or no friends	Micro
9	Neighbours contact frequency	0 = within the last day or two; 0.2 = within the last week or two; 0.4 = within the past month; 0.6 = within the past 6 months; 0.8 = within the past year; 1 = more than one year ago or no neighbours	Micro
Social Support Availability			
10	Availability of support if confined in bed	0 = all the time; 0.25 = most of the time; 0.5 = some of the time; 0.75 = a little of the time; 1 = none of the time	Micro
11	Availability of someone to talk to if needed	0 = all the time; 0.25 = most of the time; 0.5 = some of the time; 0.75 = a little of the time; 1 = none of the time	Micro
12	Availability of someone to have advice from in crisis	0 = all the time; 0.25 = most of the time; 0.5 = some of the time; 0.75 = a little of the time; 1 = none of the time	Micro
13	Availability of someone that can take to the doctor if needed	0 = all the time; 0.25 = most of the time; 0.5 = some of the time; 0.75 = a little of the time; 1 = none of the time	Micro
14	Availability from someone that shows affection	0 = all the time; 0.25 = most of the time; 0.5 = some of the time; 0.75 = a little of the time; 1 = none of the time	Micro
15	Availability of someone to have a good time	0 = all the time; 0.25 = most of the time; 0.5 = some of the time; 0.75 = a little of the time; 1 = none of the time	Micro
16	Availability from someone that helps with information	0 = all the time; 0.25 = most of the time; 0.5 = some of the time; 0.75 = a little of the time; 1 = none of the time	Meso
17	Availability of someone to confide	0 = all the time; 0.25 = most of the time; 0.5 = some of the time; 0.75 = a little of the time; 1 = none of the time	Micro
18	Availability of someone that hugs	0 = all the time; 0.25 = most of the time; 0.5 = some of the time; 0.75 = a little of the time; 1 = none of the time	Micro
19	Availability of someone to relax with	0 = all the time; 0.25 = most of the time; 0.5 = some of the time; 0.75 = a little of the time; 1 = none of the time	Micro
20	Availability of someone that prepares a meal	0 = all the time; 0.25 = most of the time; 0.5 = some of the time; 0.75 = a little of the time; 1 = none of the time	Micro
21	Availability of someone that gives wanted advice	0 = all the time; 0.25 = most of the time; 0.5 = some of the time; 0.75 = a little of the time; 1 = none of the time	Micro
22	Availability of someone to do things with	0 = all the time; 0.25 = most of the time; 0.5 = some of the time; 0.75 = a little of the time; 1 = none of the time	Micro
23	Availability of someone that helps with domestic chores	0 = all the time; 0.25 = most of the time; 0.5 = some of the time; 0.75 = a little of the time; 1 = none of the time	Micro
24	Availability of someone with whom to share fears	0 = all the time; 0.25 = most of the time; 0.5 = some of the time; 0.75 = a little of the time; 1 = none of the time	Micro
25	Availability of someone who gives suggestions	0 = all the time; 0.25 = most of the time; 0.5 = some of the time; 0.75 = a little of the time; 1 = none of the time	Micro
26	Availability of someone to do something enjoyable together	0 = all the time; 0.25 = most of the time; 0.5 = some of the time; 0.75 = a little of the time; 1 = none of the time	Micro
27	Availability of someone that understands problems	0 = all the time; 0.25 = most of the time; 0.5 = some of the time; 0.75 = a little of the time; 1 = none of the time	Micro
28	Availability of someone that makes one feel wanted	0 = all the time; 0.25 = most of the time; 0.5 = some of the time; 0.75 = a little of the time; 1 = none of the time	Micro
29	Pet owner	0 = yes; 1 = no	Micro
Social Participation			
30	Reads newspaper	0 = yes; 1 = no	Micro
31	Hobby	0 = yes; 1 = no	Micro
32	Holidays in Canada	0 = yes; 1 = no	Meso
33	Holidays outside of Canada	0 = yes; 1 = no	Meso
34	Day trip	0 = yes; 1 = no	Micro
35	Internet use	0 = yes; 1 = no	Micro
36	Voted in last election	0 = yes; 1 = no	Meso
37	Family and friends’ activities	0 = at least once a day; 0.25 = at least once a week; 0.5 = at least once a month; 0.75 = at least once a year; 1 = never	Meso
38	Sports or physical activities	0 = at least once a day; 0.25 = at least once a week; 0.5 = at least once a month; 0.75 = at least once a year; 1 = never	Micro/ Meso
39	Educational or cultural activities	0 = at least once a day; 0.25 = at least once a week; 0.5 = at least once a month; 0.75 = at least once a year; 1 = never	Micro/ Meso
40	Neighbour, community or profession activities	0 = at least once a day; 0.25 = at least once a week; 0.5 = at least once a month; 0.75 = at least once a year; 1 = never	Micro/ Meso
41	Volunteer	0 = at least once a day; 0.25 = at least once a week; 0.5 = at least once a month; 0.75 = at least once a year; 1 = never	Micro/ Meso
42	Other recreation activities	0 = at least once a day; 0.25 = at least once a week; 0.5 = at least once a month; 0.75 = at least once a year; 1 = never	Micro/ Meso
Online Social Networking			
43	Internet access	0 = yes; 1 = no	Micro
44	E-mail frequency	0 = daily; 0.25 = a few times a week; 0.5 = a few times a month; 0.75 = a few times a year; 1 = never	Micro
45	Websites frequency	0 = daily; 0.25 = a few times a week; 0.5 = a few times a month; 0.75 = a few times a year; 1 = never	Micro
46	Websites healthcare related frequency	0 = daily; 0.25 = a few times a week; 0.5 = a few times a month; 0.75 = a few times a year; 1 = never	Micro
47	Use of social networks	0 = yes; 1 = no	Micro/ Meso
48	Making friends in social networks frequency	0 = daily; 0.25 = a few times a week; 0.5 = a few times a month; 0.75 = a few times a year; 1 = never	Micro/ Meso
49	Stay in touch with friends in social networks frequency	0 = daily; 0.25 = a few times a week; 0.5 = a few times a month; 0.75 = a few times a year; 1 = never	Micro/ Meso
50	Stay in touch with family in social networks frequency	0 = daily; 0.25 = a few times a week; 0.5 = a few times a month; 0.75 = a few times a year; 1 = never	Micro/ Meso
51	Promotion in social networks frequency	0 = daily; 0.25 = a few times a week; 0.5 = a few times a month; 0.75 = a few times a year; 1 = never	Meso/ Exo
52	Other activities in social networks frequency	0 = daily; 0.25 = a few times a week; 0.5 = a few times a month; 0.75 = a few times a year; 1 = never	Micro/ Meso
Built Environment			
53	Home problems	0 = no; 1 = yes	Micro
54	Home satisfaction	0 = strongly agree; 0.33 = agree; 0.67 = disagree; 1 = strongly disagree	Micro
55	Feels part of the area	0 = strongly agree; 0.33 = agree; 0.67 = disagree; 1 = strongly disagree	Meso/ Exo
56	Vandalism	0 = strongly disagree; 0.33 = disagree; 0.67 = agree; 1 = strongly agree	Meso/ Exo
57	Feel lonely in the area	0 = strongly disagree; 0.33 = disagree; 0.67 = agree; 1 = strongly agree	Micro
58	Most people trusted in the area	0 = strongly agree; 0.33 = agree; 0.67 = disagree; 1 = strongly disagree	Meso/ Exo
59	Afraid to walk in the area	0 = strongly disagree; 0.33 = disagree; 0.67 = agree; 1 = strongly agree	Meso
60	Friendly people in the area	0 = strongly agree; 0.33 = agree; 0.67 = disagree; 1 = strongly disagree	Meso
61	People take advantage in the area	0 = strongly disagree; 0.33 = disagree; 0.67 = agree; 1 = strongly agree	Meso/ Exo
62	Clean area	0 = strongly agree; 0.33 = agree; 0.67 = disagree; 1 = strongly disagree	Meso/ Exo
63	Help available in the area	0 = strongly agree; 0.33 = agree; 0.67 = disagree; 1 = strongly disagree	Meso/ Exo
Wealth			
64	Personal income	0 = >150,000 CAD; 0.25 = 100,000–149,999 CAD; 0.5 = 50,000–99,999 CAD; 0.75 = 20,000–49,999 CAD; 1 = <20,000 CAD	Micro
65	Household income	0 = >150,000 CAD; 0.25 = 100,000–149,999 CAD; 0.5 = 50,000–99,999 CAD; 0.75 = 20,000–49,999 CAD; 1 = <20,000 CAD	Micro
66	Savings	0 = yes; 1 = no	Micro
67	Life insurance	0 = yes; 1 = no	Micro
68	Assets	0 = yes; 1 = no	Micro
69	Debts	0 = no; 1 = yes	Micro
70	Self-rated financial status	0 = manage very well; 0.2 = manage quite well; 0.4 = get by alright; 0.6 = don’t manage very well; 0.8 = have some financial difficulties; 1 = have severe financial difficulties	Micro
71	Adequate income for basic needs	0 = very well; 0.25 = adequately; 0.5 = with some difficulty; 0.75 = not very well; 1 = totally inadequately	Micro
72	Little money stops from doing things	0 = no; 1 = yes	Micro
73	Insufficient financial resources in the future	0 = little or no possibility; 0.5 = some possibility; 1 = high possibility	Micro
74	Leave inheritance	0 = high; 0.33 = moderate; 0.67 = low; 1 = none	Micro

### Characteristics of the Indices

**Fig 2:**
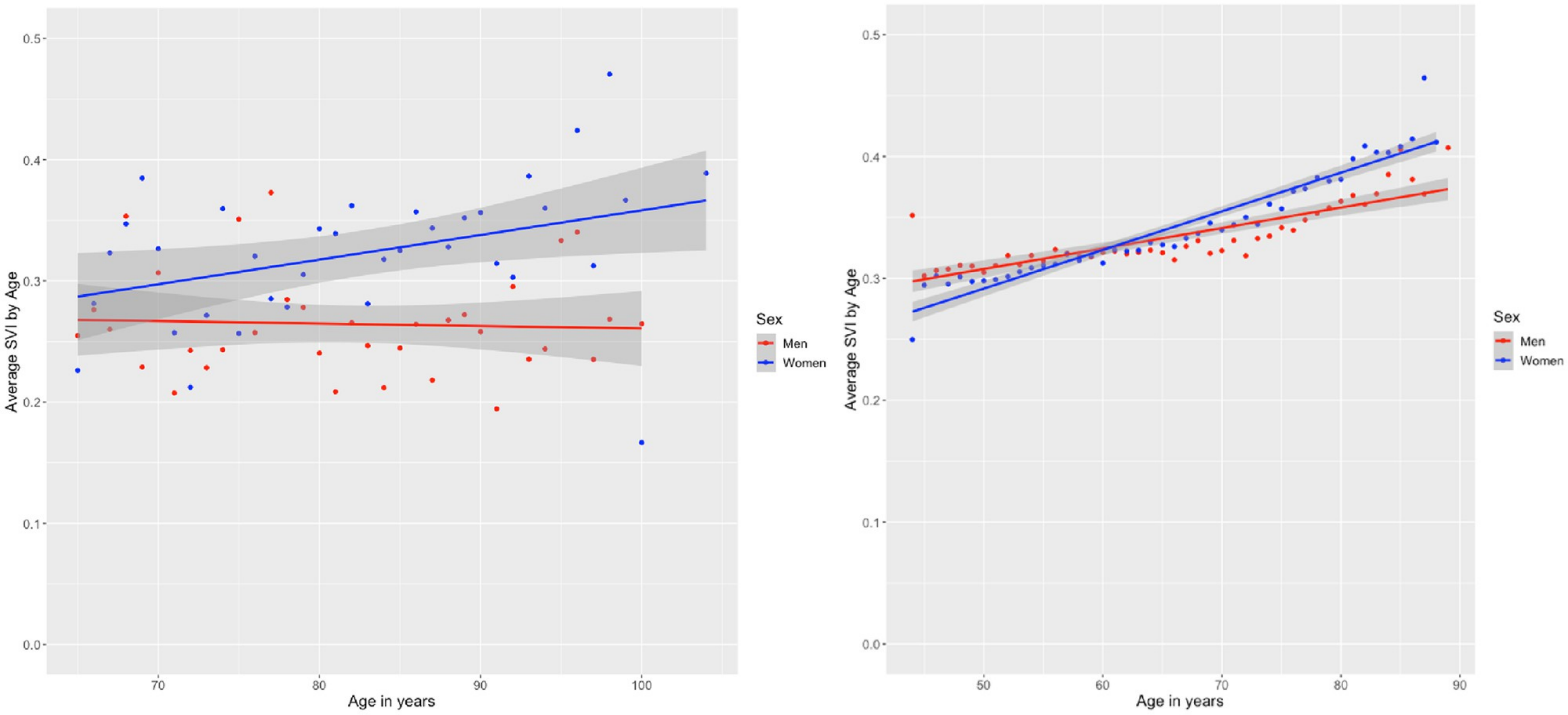
SVI by age and sex, in the SOS (left) and CLSA (right).

**Fig 3:**
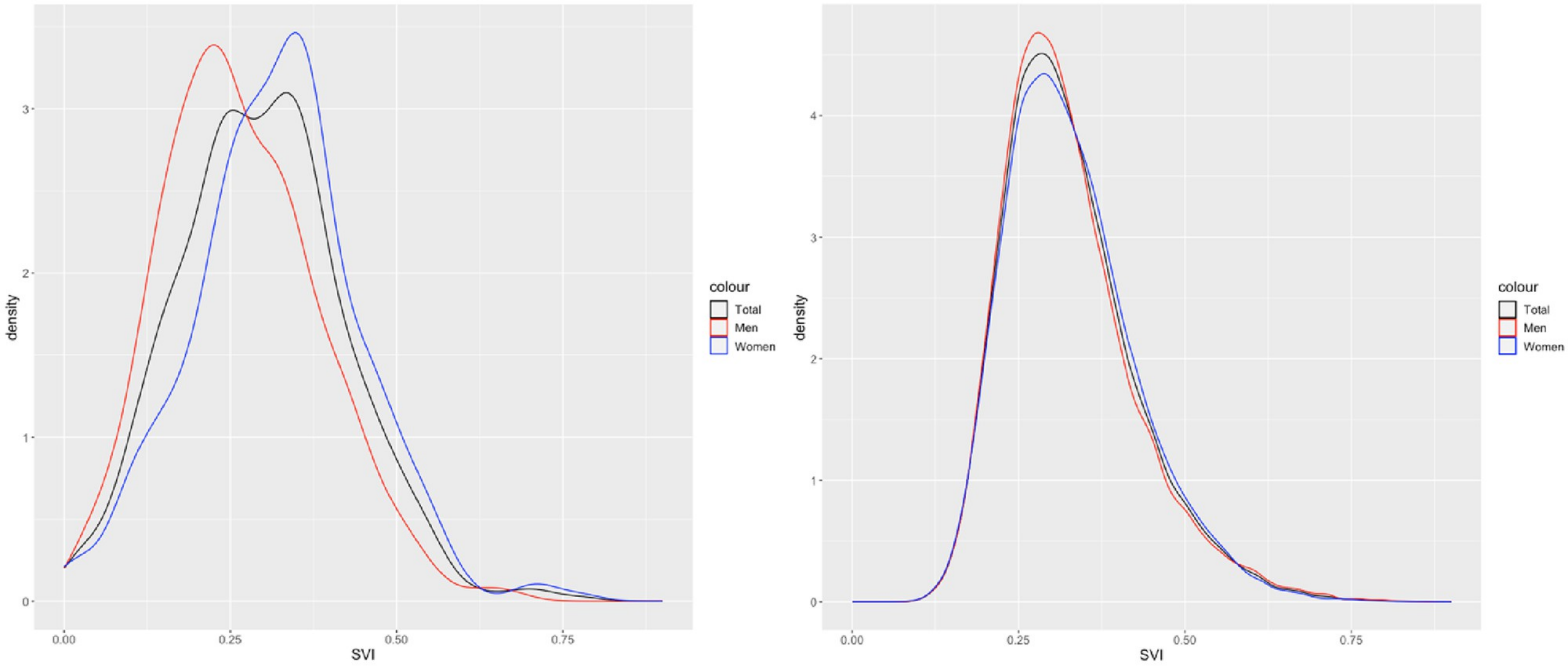
Kernel density plot of social vulnerability indices, by sex, in the SOS (left) and CSLA (right).

The mean SVI score in the SOS network dataset is 0.30(SD 0.13) and 0.22(SD 0.10) in the CLSA dataset. Descriptive statistics for the two datasets are presented in Table 3 showing that the SOS network dataset contains individuals who are older and living with a greater degree of frailty than the individuals in the CLSA cohort, which is not surprising given that all SOS participants were hospitalized. The average SVI score for women is higher on average than for men in both cohorts. Fig 2 shows the association of SVI with age by sex. In SOS, SVI increases with age only in women (r = 0.11, p = 0.04). In CLSA, SVI slowly increases by age in the total cohort (r = 0.28, p<0.001) and in men (r = 0.15, p<0.001) and women (r = 0.29, p<0.001). The distribution of the SVIs in both cohorts are shown in Fig 3. Internal consistency calculated using Cronbach’s alpha was 0.65 for the SOS SVI and 0.89 for the CLSA SVI.

**Table 3:** Characteristics of SOS and CSLA cohorts.

	SOS			CSLA		
	Total	Women	Men	Total	Women	Men
n	571	334	237	47,716*	24,332	23,384
Mean age (SD)	79.2 (8.1)	79.6 (8.1)	78.6 (8.1)	59.8 (10.3)	62.8 (10.4)**	63.2 (10.4)
Age range	65–104	65–104	65–100	45–85	45–85	45–85
SVI						
Mean	0.30 (0.13)	0.32 (0.13)**	0.29(0.12)	0.33 (0.10)	0.34 (0.10)**	0.33 (0.32)
Range	0.00–0.94	0.00–0.78	0.03–0.94	0.01–0.86	0.09–0.86	0.09–0.85
99^th^ percentile	0.64	0.66	0.60	0.62	0.61	0.63
FI						
Mean (SD)	0.20 (0.11)	0.20 (0.11)	0.20 (0.11)	0.08 (0.06)	0.09 (0.06)**	0.08 (0.05)
Range	0.00–0.62	0.01–0.62	0.00–0.57	0.00–0.54	0.00–0.51	0.00–0.54
99^th^ percentile	0.51	0.51	0.49	0.27	0.28	0.25

*12,346,610 weighted

**p < .001 t-test for differences between men vs women

### Social vulnerability and frailty and mortality

**Fig 4:**
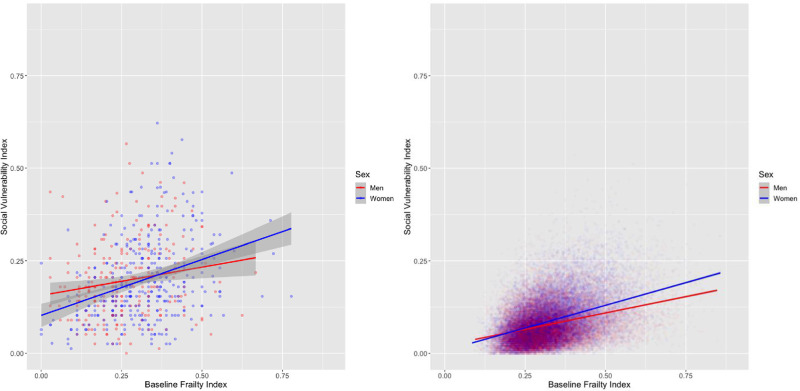
Association between SVI by FI by sex, in the SOS (left) and CLSA (right).

Frailty was measured using a 39-deficit frailty index and 52-deficit frailty index, each already validated in the SOS Network [33] and CLSA datasets [30], respectively. In the SOS, SVI is weakly correlated with frailty (r = 0.26, p<0.001). This relationship was stronger for women (r = 0.36, p<0.001) than men (r = 0.40, p<0.001) for frailty (Fig 4). In the CLSA, correlation with frailty was r = 0.37 (p<0.001) with a stronger correlation for women (r = 0.41, p<0.001) than men (r = 0.33, p<0.001). In a multivariable linear regression model adjusted for age and sex, a 0.1 increase in the FI was associated with a 0.28 (95% CI 0.23–0.42, p<0.001) increase in SVI in the SOS and a 0.68 (95% CI 0.63–0.72, p<0.001) increase in SVI in the CLSA.

**Fig 5:**
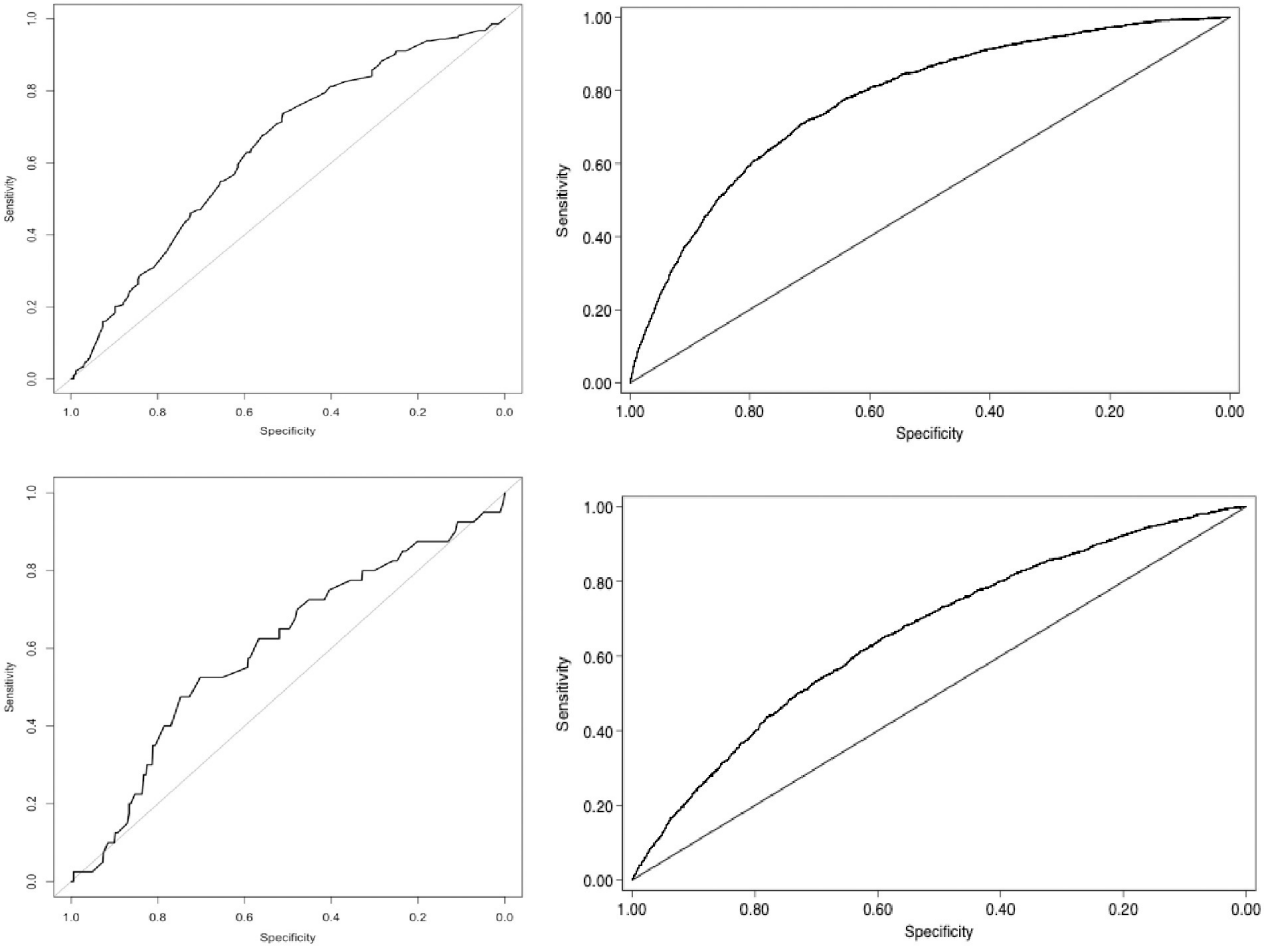
ROC curves for frailty (top panels) and mortality (bottom panels) in the SOS (left) and CLSA (right).

Fig 5 shows the ROC curves of the discrimination performance of the SVI and frailty and mortality. The AUC of the SOS SVI for baseline frailty (two weeks prior to admission) was 0.64 (95% CI: 0.59–0.69) and mortality was 0.60 (95% CI: 0.51–0.70). The AUC of the CLSA SVI was 0.77 (95% CI 0.76–0.79) for frailty and 0.66 (95% CI: 0.65–0.68) for mortality.

## Discussion

Our research group has over a decade of experience constructing SVIs. We aim to measure social vulnerability so as to capture a rich description of an individual’s social deficits (or problems), utilize data that is readily and practically measurable in population and clinical settings, respond to changing social circumstances, and predict important health outcomes.

Here we present a method of constructing a SVI that takes into consideration the whole person in society. We have highlighted the social theories underpinning the construction of this SVI and presented two examples, one in a clinical dataset with few variables and one in a larger population dataset with many variables. Here, the SVI-SOS allows us to demonstrate how to calculate the index with a smaller, homogenous population, and fewer variables. Further, we illustrate how inclusion of a SVI can be feasible with prospective clinical data collection. The SVI-CLSA provides an example of screening and selecting variables for inclusion in the index (S1 File). Furthermore, the CLSA SVI here demonstrates properties of previously calculated SVIs. Women tend to be more socially vulnerable than men, and importantly, and unlike a perfect state of health (frailty index = 0), almost no older adult has zero social vulnerability [34]. Most importantly, both SVIs are composed of deficits representing multiple domains of social circumstances and different levels of social influence from the individual to the community. In this way social vulnerability reflects how frailty can be conceptualized as being the expression of problems across multiple body systems from the cellular to organ to systems (e.g., cardiovascular) level.

Only in the last decade have SVIs become popular in the medical literature [11]. SVIs developed in non-medical fields include: Cutter et al created a SVI to environmental hazards [8], The Centres of Disease Control’s SVI initially developed for emergency management and disaster planning [4] and, from Brazil, a SVI as a tool for urban management and development [35]. All these SVIs are composed of only geographical or census level deficits. Whereas these measures subsequently were adapted to measure health outcomes, we approach social vulnerability through a health lens from the start. We especially consider the impact of social factors on the ability to resist the adverse consequences of any adverse medical event (or procedure) or to repair or cope with it. In deficit accumulation terms, the distinction between resisting a stress as “robustness” and recovery or maintenance (“resilience”) appears to be useful.

Social vulnerability measured using an index has several strengths. It gives a quantitative overview of an individual’s social circumstance, which would not be possible by examining each social variable or domain in isolation. Calculation of the SVI allows for flexibility and gradations. Small increases in social vulnerability can be captured and studied in relation to frailty and other outcomes of interest. Social vulnerability as a gradient can be useful to better differentiate risk and vulnerability. In our experience, there is value in using the SVI in tandem with the FI. Since both constructs capture heterogenous aspects of a person’s clinical picture, adding the SVI builds a better model to predict health related outcomes. This is supported by previous literature suggesting frailty indices may be strengthened by the inclusion of socioeconomic deficits [36]. Nonetheless, we view social vulnerability and frailty as distinct concepts as prior analyses show SVI is independently associated with mortality and disability even after controlling for frailty [37], and is associated with mortality in older adults without frailty [38]. Furthermore, there is great utility for policymakers and clinicians to be able to adapt or replicate the SVI in any database or population, regardless of whether the data come from surveys, clinical sources, or administrative records. The SVI can help to pinpoint vulnerable populations or regions and to target social and health resources as demonstrated by the Covid-19 pandemic [5]. Any such SVI can be constructed with a variety of social variables, so long as the basic tenant of encompassing multiple broad social domains and levels is met.

Many questions remain unanswered in this area. One limitation is that the temporal aspect of social vulnerability, or the chronosystem as described by the ecological model, remains an aspect of complex social environments not fully captured by this approach. For example, the SVI may be highly influenced by cohort effects over time–social factors that were protective in the past might not be anymore (e.g., in the CLSA reading the newspaper nowadays might be substituted by social media use, and reading the newspaper in certain cohorts may indicate a vulnerable trait). Previous work with the SVI has also suggested that social vulnerability plays a bigger role in the fittest individuals [29, 38]. Does the lack of time dependence of social vulnerability arise only when the frailest have already died? In comparison to our work on frailty, social vulnerability is not as strongly associated with age. Future research is encouraged to examine the dynamics of social vulnerability, and how they change with age, and whether they show temporal trends. Additionally, we build our SVI using a deficit accumulation approach, but is this the same as taking a resilience approach? Social vulnerability should ideally encompass two concepts articulated by Ukraintseva, Yashin and Arbeev: robustness (the ability to resist deviation from the healthier state) and resilience (recovery to the healthier state aided by a well connected and supportive social situation) [39]. Our SVI does not distinguish whether the absence of a deficit is the same as the presence of a resilience factor. Conceivably these would not confer the same degree of (dis)advantage yet only the former is captured by our index. Such considerations are motivating additional inquires by our group.

Another area for further research surrounds the measurement properties of SVIs. Further work should continue to evaluate validity and reliability of these indices. SVIs may have features of both formative and reflective models, however, future research may also focus on replicating features of the deficit accumulation approach seen in the frailty index [40]. Frailty indices, even if they do not consist of the same items or use the same databases, show similar accumulation patterns (i.e. 0.03/year) and have a maximal limit accumulation [24, 41]. A limitation of this paper is the varied number of deficits in the SVI–the SOS may have too few items and the CLSA SVI may have redundant items. Determining if SVI performance is similar after a certain number of items is another area of exploration.

## Conclusion

We present a standard method of constructing a SVI. In our holistic approach to understanding social circumstances, our SVI incorporates factors from multiple social domains and levels in a social-ecological model. We demonstrate construction of the SVI and its feasibility in two different datasets, with the potential for operationalization in many other datasets. Social vulnerability may have reproducible associations with age, sex and frailty. This SVI can be used to improve our understanding of social vulnerability and its impacts on the health of communities and individuals.

## Supporting information

S1 File (DOCX)
