# Supplementary material for: A standard procedure for constructing a multi-level social vulnerability index using CLSA and SOS data as working examples
Source: PLoS One. 2024 Dec 13;19(12):e0315474. doi: 10.1371/journal.pone.0315474 (PMC11642991; doi:10.1371/journal.pone.0315474)
Supplement: S1 File — (DOCX) [file pone.0315474.s001.docx]

**Supporting Information 1**

Screening SVI variables in the CLSA

| # | CLSA Domain | Item | Categories | Coding | Missing % | Weighted values | | | | |
| --- | --- | --- | --- | --- | --- | --- | --- | --- | --- | --- |
|  |  |  |  |  |  | % | % 95% CI | | N | Total |
|  |  |  |  |  |  |  | Lower bound | Upper bound |  |  |
| 1 | Socio-Demographic | Marital status | Yes | 0 | 0.1 | 75.78 | 75.04 | 76.51 | 10,347,052 | 13,650,465 |
|  |  |  | No | 1 |  | 24.21 | 23.48 | 24.95 | 3,303,413 |  |
| 2 | Home Ownership | Home owner | Yes | 0 | 0.2 | 85.64 | 84.99 | 86.27 | 11,659,200 | 13,620,561 |
|  |  |  | No | 1 |  | 14.35 | 13.72 | 15 | 1,961,361 |  |
| 3 | Education | Education | College, university bachelor, graduate, or professional degree | 0 | 0.2 | 41.92 | 41.07 | 42.77 | 5,719,170 | 13,643,058 |
|  |  |  | Trades or apprenticeship | 0.33 |  | 13.46 | 12.88 | 14.05 | 1,836,356 |  |
|  |  |  | High school | 0.66 |  | 22.66 | 21.94 | 23.4 | 3,091,517 |  |
|  |  |  | Less than high school | 1 |  | 21.96 | 21 | 22.96 | 2,996,016 |  |
| 4 | Social Networks | Living alone | No | 0 | 0.1 | 84.35 | 83.79 | 84.9 | 11,521,078 | 13,650,566 |
|  |  |  | Yes | 1 |  | 15.65 | 15.10 | 16.21 | 2,129,488 |  |
| 5 |  | Child contact frequency | Within the last day or two or all children live in household | 0 | 0.1 | 40.96 | 40.05 | 41.87 | 5,580,322 | 13,643,820 |
|  |  |  | Within the last week or two | 0.2 |  | 28.02 | 27.21 | 28.84 | 3,833,913 |  |
|  |  |  | Within the past month | 0.4 |  | 8.43 | 7.96 | 8.93 | 1,146,081 |  |
|  |  |  | Within the past 6 months | 0.6 |  | 6.7 | 6.37 | 7.26 | 927,780 |  |
|  |  |  | Within the past year | 0.8 |  | 1.06 | 0.92 | 1.22 | 150,082 |  |
|  |  |  | More than 1 year ago or no children | 1 |  | 14.73 | 14.11 | 15.38 | 2,005,642 |  |
| 6 |  | Siblings contact frequency | Within the last day or two or all siblings live in household | 0 | 0.1 | 11.51 | 10.93 | 12.11 | 1,569,018 | 13,643,631 |
|  |  |  | Within the last week or two | 0.2 |  | 24.95 | 24.14 | 25.77 | 4,079,446 |  |
|  |  |  | Within the past month | 0.4 |  | 15.81 | 15.14 | 16.49 | 2,155,694 |  |
|  |  |  | Within the past 6 months | 0.6 |  | 21.56 | 20.84 | 22.31 | 2,933,381 |  |
|  |  |  | Within the past year | 0.8 |  | 6.55 | 6.11 | 7.01 | 886,836 |  |
|  |  |  | More than 1 year ago or no siblings | 1 |  | 19.63 | 18.93 | 20.34 | 2,674,152 |  |
| 7 |  | Relatives contact frequency | Within the last day or two or all relatives live in household | 0 | 0.2 | 14.68 | 14.03 | 15.36 | 2,002,766 | 13,624,260 |
|  |  |  | Within the last week or two | 0.2 |  | 25.76 | 24.98 | 26.56 | 3,515,059 |  |
|  |  |  | Within the past month | 0.4 |  | 14.72 | 14.1 | 15.37 | 2,002,766 |  |
|  |  |  | Within the past 6 months | 0.6 |  | 21.18 | 20.45 | 21.94 | 2,888,343 |  |
|  |  |  | Within the past year | 0.8 |  | 7.46 | 6.97 | 7.98 | 1,021,820 |  |
|  |  |  | More than 1 year ago or no relatives | 1 |  | 16.19 | 15.52 | 16.87 | 2,207,130 |  |
| 8 |  | Friends contact frequency | Within the last day or two or all friends live in household | 0 | 0.1 | 31.51 | 30.66 | 32.37 | 4,293,872 | 13,631,339 |
|  |  |  | Within the last week or two | 0.2 |  | 39.24 | 38.35 | 40.14 | 5,343,485 |  |
|  |  |  | Within the past month | 0.4 |  | 11.03 | 10.51 | 11.59 | 1,499,447 |  |
|  |  |  | Within the past 6 months | 0.6 |  | 7.92 | 7.44 | 8.43 | 1,076,876 |  |
|  |  |  | Within the past year | 0.8 |  | 1.37 | 1.18 | 1.59 | 190,839 |  |
|  |  |  | More than 1 year ago or no friends | 1 |  | 8.92 | 8.39 | 9.47 | 1,213,189 |  |
| 9 |  | Neighbours contact frequency | Within the last day or two | 0 | 5.5 | 23.72 | 22.95 | 24.52 | 3,038,741 | 12,821,690 |
|  |  |  | Within the last week or two | 0.2 |  | 23.83 | 23.06 | 24.62 | 3,051,562 |  |
|  |  |  | Within the past month | 0.4 |  | 9.39 | 8.85 | 9.96 | 1,205,239 |  |
|  |  |  | Within the past 6 months | 0.6 |  | 10.3 | 9.73 | 10.9 | 1,320,634 |  |
|  |  |  | Within the past year | 0.8 |  | 3.39 | 3.02 | 3.79 | 435,937 |  |
|  |  |  | More than 1 year ago or no neighbors | 1 |  | 29.36 | 28.5 | 30.24 | 3,769,577 |  |
| 10 | Social Support Availability | Availability of support if confined in bed | All the time | 0 | 0.8 | 51.74 | 50.83 | 52.66 | 7,008,711 | 13,546,020 |
|  |  |  | Most of the time | 0.25 |  | 26.08 | 25.3 | 26.87 | 3,532,802 |  |
|  |  |  | Some of the time | 0.5 |  | 13.53 | 12.94 | 14.14 | 1,832,777 |  |
|  |  |  | A little of the time | 0.75 |  | 4.7 | 4.32 | 5.11 | 636,663 |  |
|  |  |  | None of the time | 1 |  | 3.95 | 3.6 | 4.34 | 535,068 |  |
| 11 |  | Availability of someone to talk to if needed | All the time | 0 | 0.2 | 60.82 | 59.94 | 61.7 | 8,285,796 | 13,623,472 |
|  |  |  | Most of the time | 0.25 |  | 26.77 | 25.99 | 27.56 | 3,621,119 |  |
|  |  |  | Some of the time | 0.5 |  | 8.15 | 7.7 | 8.64 | 1,542,177 |  |
|  |  |  | A little of the time | 0.75 |  | 2.89 | 2.58 | 3.24 | 505,431 |  |
|  |  |  | None of the time | 1 |  | 1.36 | 1.17 | 1.58 | 498,619 |  |
| 12 |  | Availability of someone to have advice from in crisis | All the time | 0 | 0.6 | 54.73 | 53.82 | 55.63 | 7,419,625 | 13,556,779 |
|  |  |  | Most of the time | 0.25 |  | 26.58 | 25.79 | 27.39 | 3,603,392 |  |
|  |  |  | Some of the time | 0.5 |  | 11.32 | 10.79 | 11.88 | 1,534,627 |  |
|  |  |  | A little of the time | 0.75 |  | 3.71 | 3.4 | 4.04 | 502,957 |  |
|  |  |  | None of the time | 1 |  | 3.66 | 3.31 | 4.04 | 496,178 |  |
| 13 |  | Availability of someone that can take to the doctor if needed | All the time | 0 | 0.4 | 67.40 | 66.56 | 68.22 | 9,170,059 | 13,605,429 |
|  |  |  | Most of the time | 0.25 |  | 20.70 | 20.01 | 21.41 | 2,816,324 |  |
|  |  |  | Some of the time | 0.5 |  | 7.26 | 6.81 | 7.73 | 987,754 |  |
|  |  |  | A little of the time | 0.75 |  | 2.37 | 2.12 | 2.65 | 322,449 |  |
|  |  |  | None of the time | 1 |  | 2.27 | 2 | 2.58 | 322,449 |  |
| 14 |  | Availability from someone that shows affection | All the time | 0 | 0.2 | 76.20 | 75.45 | 76.94 | 10,383,842 | 13,627,089 |
|  |  |  | Most of the time | 0.25 |  | 15.56 | 14.94 | 16.21 | 2,120,375 |  |
|  |  |  | Some of the time | 0.5 |  | 5.22 | 4.87 | 5.58 | 711,334 |  |
|  |  |  | A little of the time | 0.75 |  | 1.74 | 1.51 | 2 | 237,111 |  |
|  |  |  | None of the time | 1 |  | 1.28 | 1.10 | 1.48 | 174,427 |  |
| 15 |  | Availability of someone to have a good time | All the time | 0 | 0.3 | 61.22 | 60.34 | 62.1 | 8,340,772 | 13,624,260 |
|  |  |  | Most of the time | 0.25 |  | 25.36 | 24.58 | 26.16 | 3,455,112 |  |
|  |  |  | Some of the time | 0.5 |  | 10.06 | 9.56 | 10.59 | 1,370,601 |  |
|  |  |  | A little of the time | 0.75 |  | 2 | 1.79 | 2.24 | 272,485 |  |
|  |  |  | None of the time | 1 |  | 1.35 | 1.15 | 1.58 | 183,928 |  |
| 16 |  | Availability from someone that helps with information | All the time | 0 | 0.5 | 54.01 | 53.10 | 54.92 | 7,331,283 | 13,573,936 |
|  |  |  | Most of the time | 0.25 |  | 30.17 | 29.35 | 31.01 | 4,095,256 |  |
|  |  |  | Some of the time | 0.5 |  | 11.42 | 10.88 | 11.98 | 1,550,143 |  |
|  |  |  | A little of the time | 0.75 |  | 2.77 | 2.47 | 3.1 | 375,998 |  |
|  |  |  | None of the time | 1 |  | 1.63 | 1.42 | 1.86 | 221,255 |  |
| 17 |  | Availability of someone to confide | All the time | 0 | 0.4 | 57.65 | 56.76 | 58.55 | 7,842,880 | 13,604,301 |
|  |  |  | Most of the time | 0.25 |  | 25.61 | 24.84 | 26.39 | 3,484,061 |  |
|  |  |  | Some of the time | 0.5 |  | 10.72 | 10.19 | 11.27 | 1,458,381 |  |
|  |  |  | A little of the time | 0.75 |  | 3.82 | 3.49 | 4.17 | 519,140 |  |
|  |  |  | None of the time | 1 |  | 2.2 | 1.94 | 2.51 | 299,703 |  |
| 18 |  | Availability of someone that hugs | All the time | 0 | 0.3 | 63.59 | 62.72 | 64.44 | 8,658,767 | 13,616,555 |
|  |  |  | Most of the time | 0.25 |  | 19.48 | 18.78 | 20.19 | 2,652,505 |  |
|  |  |  | Some of the time | 0.5 |  | 10.07 | 9.58 | 10.59 | 1,371,187 |  |
|  |  |  | A little of the time | 0.75 |  | 4.19 | 3.85 | 4.56 | 570,670 |  |
|  |  |  | None of the time | 1 |  | 2.68 | 2.4 | 2.98 | 364,243 |  |
| 19 |  | Availability of someone to relax with | All the time | 0 | 0.4 | 51.54 | 50.63 | 52.46 | 7,014,082 | 13,609,007 |
|  |  |  | Most of the time | 0.25 |  | 28.61 | 27.81 | 29.42 | 3,893,537 |  |
|  |  |  | Some of the time | 0.5 |  | 14.29 | 13.7 | 14.9 | 1,944,727 |  |
|  |  |  | A little of the time | 0.75 |  | 3.43 | 3.12 | 3.78 | 467,333 |  |
|  |  |  | None of the time | 1 |  | 2.13 | 1.87 | 2.42 | 289,464 |  |
| 20 |  | Availability of someone that prepares a meal | All the time | 0 | 0.7 | 58.11 | 57.23 | 59 | 7,880,108 | 13,560,675 |
|  |  |  | Most of the time | 0.25 |  | 22.06 | 21.35 | 22.79 | 2,991,485 |  |
|  |  |  | Some of the time | 0.5 |  | 11.70 | 11.18 | 12.25 | 1,586,599 |  |
|  |  |  | A little of the time | 0.75 |  | 4.47 | 4.13 | 4.84 | 606,433 |  |
|  |  |  | None of the time | 1 |  | 3.65 | 3.34 | 3.99 | 495,100 |  |
| 21 |  | Availability of someone that gives wanted advice | All the time | 0 | 0.7 | 45.87 | 44.96 | 46.79 | 6,211,864 | 13,542,325 |
|  |  |  | Most of the time | 0.25 |  | 28.21 | 27.41 | 29.01 | 3,820,290 |  |
|  |  |  | Some of the time | 0.5 |  | 16.89 | 16.23 | 17.56 | 2,287,299 |  |
|  |  |  | A little of the time | 0.75 |  | 5.44 | 5.04 | 5.87 | 736,432 |  |
|  |  |  | None of the time | 1 |  | 3.6 | 3.26 | 3.97 | 486,982 |  |
| 22 |  | Availability of someone to do things with | All the time | 0 | 0.6 | 44.57 | 43.65 | 45.49 | 6,054,918 | 13,585,188 |
|  |  |  | Most of the time | 0.25 |  | 30.62 | 29.81 | 31.45 | 4,159,785 |  |
|  |  |  | Some of the time | 0.5 |  | 18.09 | 17.42 | 18.79 | 2,457,561 |  |
|  |  |  | A little of the time | 0.75 |  | 4.2 | 3.88 | 4.55 | 570,850 |  |
|  |  |  | None of the time | 1 |  | 2.51 | 2.23 | 2.84 | 341,396 |  |
| 23 |  | Availability of someone that helps with domestic chores | All the time | 0 | 0.6 | 51.79 | 50.87 | 52.7 | 7,031,118 | 13,576,207 |
|  |  |  | Most of the time | 0.25 |  | 26.27 | 25.48 | 27.08 | 3,566,470 |  |
|  |  |  | Some of the time | 0.5 |  | 14.34 | 13.75 | 14.96 | 1,946,828 |  |
|  |  |  | A little of the time | 0.75 |  | 4.64 | 4.3 | 5 | 629,800 |  |
|  |  |  | None of the time | 1 |  | 2.96 | 2.66 | 3.28 | 401,448 |  |
| 24 |  | Availability of someone with whom to share fears | All the time | 0 | 0.7 | 53.26 | 52.35 | 54.17 | 7,225,053 | 13,565,628 |
|  |  |  | Most of the time | 0.25 |  | 24.41 | 23.65 | 25.19 | 3,311,370 |  |
|  |  |  | Some of the time | 0.5 |  | 12.68 | 12.12 | 13.26 | 1,720,122 |  |
|  |  |  | A little of the time | 0.75 |  | 5.22 | 4.86 | 5.61 | 708,668 |  |
|  |  |  | None of the time | 1 |  | 4.42 | 4.05 | 4.83 | 600,143 |  |
| 25 |  | Availability of someone who gives suggestions | All the time | 0 | 0.5 | 50.45 | 49.53 | 51.36 | 6,852,124 | 13,582,009 |
|  |  |  | Most of the time | 0.25 |  | 28.48 | 27.67 | 29.3 | 3,868,156 |  |
|  |  |  | Some of the time | 0.5 |  | 14.18 | 13.58 | 14.8 | 1,925,929 |  |
|  |  |  | A little of the time | 0.75 |  | 4.24 | 3.89 | 4.62 | 576,013 |  |
|  |  |  | None of the time | 1 |  | 2.66 | 2.38 | 2.96 | 360,602 |  |
| 26 |  | Availability of someone to do something enjoyable together | All the time | 0 | 0.3 | 53.9 | 52.99 | 54.8 | 7,338,401 | 13,614,845 |
|  |  |  | Most of the time | 0.25 |  | 30.38 | 29.57 | 31.2 | 4,136,190 |  |
|  |  |  | Some of the time | 0.5 |  | 12.44 | 11.88 | 13.03 | 1,693,687 |  |
|  |  |  | A little of the time | 0.75 |  | 2.06 | 1.83 | 2.32 | 280,602 |  |
|  |  |  | None of the time | 1 |  | 1.22 | 1.01 | 1.46 | 165,557 |  |
| 27 |  | Availability of someone that understands problems | All the time | 0 | 0.7 | 47.29 | 46.37 | 48.21 | 6,408,040 | 13,550,518 |
|  |  |  | Most of the time | 0.25 |  | 31.82 | 31 | 32.65 | 4,311,775 |  |
|  |  |  | Some of the time | 0.5 |  | 15.02 | 14.4 | 15.66 | 2,035,288 |  |
|  |  |  | A little of the time | 0.75 |  | 3.58 | 3.27 | 3.92 | 485,244 |  |
|  |  |  | None of the time | 1 |  | 2.28 | 2 | 2.61 | 309,358 |  |
| 28 |  | Availability of someone that makes one feel wanted | All the time | 0 | 0.4 | 67.1 | 66.24 | 67.92 | 9,127,896 | 13,607,477 |
|  |  |  | Most of the time | 0.25 |  | 20.45 | 19.74 | 21.17 | 2,782,729 |  |
|  |  |  | Some of the time | 0.5 |  | 7.60 | 7.16 | 8.06 | 1,033,760 |  |
|  |  |  | A little of the time | 0.75 |  | 2.75 | 2.48 | 3.06 | 374,614 |  |
|  |  |  | None of the time | 1 |  | 2.12 | 1.88 | 2.38 | 287,934 |  |
| 29 |  | Pet owner | Yes | 0 | 0.3 | 50.3 | 49.38 | 51.21 | 6,854,561 | 13,627,357 |
|  |  |  | No | 1 |  | 49.7 | 48.79 | 50.62 | 6,772,796 |  |
| 30 | Social Participation | Reads newspaper | Yes | 0 | 0.1 | 57.25 | 56.33 | 58.17 | 7,814,949 | 13,650,566 |
|  |  |  | No | 1 |  | 42.75 | 41.83 | 43.67 | 5,835,617 |  |
| 31 |  | Hobby | Yes | 0 | 0.1 | 90.11 | 89.50 | 90.69 | 12,300,525 | 13,650,566 |
|  |  |  | No | 1 |  | 9.89 | 9.31 | 10.50 | 1,349,904 |  |
| 32 |  | Holidays in Canada | Yes | 0 | 0.1 | 65.93 | 65.04 | 66.81 | 8,999,818 | 13,650,566 |
|  |  |  | No | 1 |  | 34.07 | 33.19 | 34.96 | 4,650,748 |  |
| 33 |  | Holidays outside of Canada | Yes | 0 | 0.1 | 51.32 | 50.41 | 52.24 | 7,005,470 | 13,650,566 |
|  |  |  | No | 1 |  | 48.68 | 47.76 | 49.59 | 6,645,096 |  |
| 34 |  | Day trip | Yes | 0 | 0.1 | 90.7 | 90.17 | 91.21 | 12,381,063 | 13,650,566 |
|  |  |  | No | 1 |  | 9.3 | 8.79 | 9.83 | 1,269,776 |  |
| 35 |  | Internet use | Yes | 0 | 0.1 | 85.25 | 84.52 | 85.95 | 11,637,108 | 13,650,566 |
|  |  |  | No | 1 |  | 14.75 | 14.05 | 15.48 | 2,013,458 |  |
| 36 |  | Voted in last election | Yes | 0 | 0.1 | 91.82 | 91.25 | 92.36 | 12,533,950 | 13,650,566 |
|  |  |  | No | 1 |  | 8.18 | 7.64 | 8.75 | 1,116,616 |  |
| 37 |  | Family and friends’ activities | At least once a day | 0 | 0.1 | 3.8 | 3.46 | 4.17 | 517,695 | 13,641,496 |
|  |  |  | At least once a week | 0.25 |  | 43.99 | 43.09 | 44.89 | 6,000,894 |  |
|  |  |  | At least once a month | 0.5 |  | 39.97 | 39.07 | 40.88 | 5,452,506 |  |
|  |  |  | At least once a year | 0.75 |  | 10.47 | 9.88 | 11.08 | 1,428,265 |  |
|  |  |  | Never | 1 |  | 1.78 | 1.54 | 2.05 | 242,682 |  |
| 38 |  | Sports or physical activities | At least once a day | 0 | 0.2 | 7.9 | 7.42 | 8.41 | 1,077,563 | 13,638,309 |
|  |  |  | At least once a week | 0.25 |  | 39.95 | 39.07 | 40.83 | 5,448,504 |  |
|  |  |  | At least once a month | 0.5 |  | 15.31 | 14.68 | 15.97 | 2,088,025 |  |
|  |  |  | At least once a year | 0.75 |  | 7.4 | 6.9 | 7.93 | 1,008,962 |  |
|  |  |  | Never | 1 |  | 29.44 | 28.59 | 30.31 | 4,015,118 |  |
| 39 |  | Educational or cultural activities | At least once a day | 0 | 0.1 | 0.63 | 0.5 | 0.8 | 86,073 | 13,640,666 |
|  |  |  | At least once a week | 0.25 |  | 7.6 | 7.19 | 8.03 | 1,036,691 |  |
|  |  |  | At least once a month | 0.5 |  | 33.44 | 32.61 | 34.27 | 4,561,439 |  |
|  |  |  | At least once a year | 0.75 |  | 35.21 | 34.34 | 36.09 | 4,802,878 |  |
|  |  |  | Never | 1 |  | 23.12 | 22.28 | 23.99 | 3,153,722 |  |
| 40 |  | Neighbor, community, or profession activities | At least once a day | 0 | 0.3 | 0.63 | 0.52 | 0.77 | 86,410 | 13,627,184 |
|  |  |  | At least once a week | 0.25 |  | 6.98 | 6.57 | 7.41 | 951,041 |  |
|  |  |  | At least once a month | 0.5 |  | 16.69 | 16.07 | 17.34 | 2,274,377 |  |
|  |  |  | At least once a year | 0.75 |  | 19.88 | 19.18 | 20.59 | 2,709,084 |  |
|  |  |  | Never | 1 |  | 55.82 | 54.92 | 56.71 | 7,606,694 |  |
| 41 |  | Volunteer | At least once a day | 0 | 0.2 | 1.50 | 1.33 | 1.70 | 205,095 | 13,636,611 |
|  |  |  | At least once a week | 0.25 |  | 14.49 | 13.90 | 15.11 | 1,975,945 |  |
|  |  |  | At least once a month | 0.5 |  | 17.87 | 17.22 | 18.54 | 2,436,862 |  |
|  |  |  | At least once a year | 0.75 |  | 20.15 | 19.44 | 20.88 | 2,747,777 |  |
|  |  |  | Never | 1 |  | 45.98 | 45.07 | 46.90 | 6,270,114 |  |
| 42 |  | Other recreation activities | At least once a day | 0 | 0.1 | 4.25 | 3.88 | 4.66 | 525,523 | 12,359,438 |
|  |  |  | At least once a week | 0.25 |  | 42.45 | 41.52 | 43.39 | 5,246,581 |  |
|  |  |  | At least once a month | 0.5 |  | 25.03 | 24.22 | 25.85 | 3,093,567 |  |
|  |  |  | At least once a year | 0.75 |  | 12.7 | 12.04 | 13.4 | 1,569,649 |  |
|  |  |  | Never | 1 |  | 15.56 | 14.88 | 16.28 | 1,923,129 |  |
| 43 | Income | Personal income | ≥150,000 CAD | 0 | 4.2 | 3.42 | 3.13 | 3.74 | 445,716 | 13,028,831 |
|  |  |  | 100,000-149,9999 CAD | 0.25 |  | 6.64 | 6.23 | 7.08 | 865,766 |  |
|  |  |  | 50,000-9,999 CAD | 0.5 |  | 28.56 | 27.74 | 29.4 | 3,721,034 |  |
|  |  |  | 20,000-49,999 CAD | 0.75 |  | 28.95 | 38.04 | 39.87 | 5,074,730 |  |
|  |  |  | <20,000 CAD | 1 |  | 22.42 | 21.59 | 23.28 | 2,921,064 |  |
| 44 |  | Household income | ≥150,000 CAD | 0 | 5.3 | 13.31 | 12.71 | 13.94 | 1,718,704 | 12,912,874 |
|  |  |  | 100,000-149,9999 CAD | 0.25 |  | 17.60 | 16.91 | 18.32 | 2,272,666 |  |
|  |  |  | 50,000-9,999 CAD | 0.5 |  | 35.73 | 34.82 | 36.64 | 4,613,770 |  |
|  |  |  | 20,000-49,999 CAD | 0.75 |  | 26.56 | 25.72 | 27.41 | 3,429,659 |  |
|  |  |  | <20,000 CAD | 1 |  | 6.80 | 6.33 | 7.30 | 877,946 |  |
| 45 | Online Social Networking | Internet access | Yes | 0 | 0.2 | 89.40 | 90.03 | 11.27 | 11,027,309 | 12,334,797 |
|  |  |  | No | 1 |  | 10.60 | 9.97 | 11.27 | 1,307,488 |  |
| 46 |  | E-mail frequency | Daily | 0 | 0.3 | 65.18 | 64.23 | 66.11 | 8,032,614 | 12,323,740 |
|  |  |  | A few times a week | 0.25 |  | 13.37 | 12.72 | 14.04 | 1,647,684 |  |
|  |  |  | A few times a month | 0.5 |  | 4.56 | 4.15 | 5.01 | 561,470 |  |
|  |  |  | A few times a year | 0.75 |  | 1.37 | 1.17 | 1.6 | 168,466 |  |
|  |  |  | Never | 1 |  | 15.33 | 14.78 | 16.32 | 1,913,877 |  |
| 47 |  | Websites frequency | Daily | 0 | 0.3 | 61.25 | 60.3 | 62.19 | 7,548,870 | 12,324,685 |
|  |  |  | A few times a week | 0.25 |  | 17.81 | 17.09 | 18.56 | 2,195,026 |  |
|  |  |  | A few times a month | 0.5 |  | 5.44 | 5.03 | 5.89 | 670,956 |  |
|  |  |  | A few times a year | 0.75 |  | 2.05 | 1.73 | 2.42 | 252,040 |  |
|  |  |  | Never | 1 |  | 13.45 | 12.77 | 14.16 | 1,657,670 |  |
| 48 |  | Websites health related frequency | Daily | 0 | 0.5 | 4.13 | 3.79 | 4.50 | 508,251 | 12,303,351 |
|  |  |  | A few times a week | 0.25 |  | 13.63 | 12.97 | 14.31 | 1,676,947 |  |
|  |  |  | A few times a month | 0.5 |  | 28.94 | 28.11 | 29.79 | 3,560,590 |  |
|  |  |  | A few times a year | 0.75 |  | 27.70 | 26.87 | 28.55 | 3,408,028 |  |
|  |  |  | Never | 1 |  | 25.59 | 24.73 | 26.48 | 3,148,428 |  |
| 49 |  | Use of social networks | Yes | 0 | 0.3 | 49.21 | 48.25 | 50.16 | 6,071,626 | 12,338,195 |
|  |  |  | No | 1 |  | 50.79 | 49.84 | 51.75 | 6,266,569 |  |
| 50 |  | Making friends in social networks frequency | Daily | 0 | 0.3 | 0.92 | 0.67 | 1.25 | 113,307 | 12,336,044 |
|  |  |  | A few times a week | 0.25 |  | 0.66 | 0.51 | 0.85 | 80,801 |  |
|  |  |  | A few times a month | 0.5 |  | 0.77 | 0.62 | 0.95 | 94,420 |  |
|  |  |  | A few times a year | 0.75 |  | 1 | 0.77 | 1.29 | 122,842 |  |
|  |  |  | Never | 1 |  | 96.67 | 96.19 | 97.08 | 11,925,254 |  |
| 51 |  | Stay in touch with friends in social networks frequency | Daily | 0 | 0.4 | 18.62 | 17.87 | 19.38 | 2,295,219 | 12,326,632 |
|  |  |  | A few times a week | 0.25 |  | 11.85 | 11.26 | 12.47 | 1,460,706 |  |
|  |  |  | A few times a month | 0.5 |  | 5.33 | 4.93 | 5.76 | 657,256 |  |
|  |  |  | A few times a year | 0.75 |  | 1.50 | 1.28 | 1.75 | 184,776 |  |
|  |  |  | Never | 1 |  | 62.70 | 61.77 | 63.62 | 7,728,798 |  |
| 52 |  | Stay in touch with family in social networks frequency | Daily | 0 | 0.4 | 17.51 | 16.79 | 18.26 | 2,157,460 | 12,321,300 |
|  |  |  | A few times a week | 0.25 |  | 11.87 | 11.28 | 12.49 | 1,462,538 |  |
|  |  |  | A few times a month | 0.5 |  | 5.58 | 5.17 | 6.03 | 687,652 |  |
|  |  |  | A few times a year | 0.75 |  | 1.97 | 1.72 | 2.25 | 242,360 |  |
|  |  |  | Never | 1 |  | 63.07 | 62.14 | 63.99 | 7,771,044 |  |
| 53 |  | Promotion in social networks frequency | Daily | 0 | 0.2 | 2.74 | 2.46 | 3.05 | 338,110 | 12,335,267 |
|  |  |  | A few times a week | 0.25 |  | 2.47 | 2.17 | 2.80 | 304,311 |  |
|  |  |  | A few times a month | 0.5 |  | 2.47 | 2.17 | 2.80 | 335,643 |  |
|  |  |  | A few times a year | 0.75 |  | 1.20 | 0.95 | 1.51 | 148,023 |  |
|  |  |  | Never | 1 |  | 90.87 | 90.29 | 91.42 | 11,200,422 |  |
| 54 |  | Other activities in social networks frequency | Daily | 0 | 0.3 | 3.22 | 2.92 | 3.54 | 397,092 | 12,335,889 |
|  |  |  | A few times a week | 0.25 |  | 1.33 | 1.15 | 1.53 | 163,821 |  |
|  |  |  | A few times a month | 0.5 |  | 0.59 | 0.48 | 0.74 | 73,090 |  |
|  |  |  | A few times a year | 0.75 |  | 0.20 | 0.14 | 0.37 | 27,768 |  |
|  |  |  | Never | 1 |  | 94.63 | 94.23 | 95.02 | 11,673,452 |  |
| 55 | Built Environments | Home problems | No | 0 | 0.1 | 78.58 | 77.80 | 79.34 | 9,712,785 | 12,360,378 |
|  |  |  | Yes | 1 |  | 21.42 | 20.66 | 22.20 | 2,647,593 |  |
| 56 |  | Home satisfaction | Strongly agree | 0 | 0.5 | 64.34 | 63.42 | 65.25 | 7,913,527 | 12,299,544 |
|  |  |  | Agree | 0.33 |  | 31.46 | 30.57 | 32.36 | 3,869,437 |  |
|  |  |  | Disagree | 0.66 |  | 3.29 | 2.97 | 3.65 | 404,778 |  |
|  |  |  | Strongly disagree | 1 |  | 0.90 | 0.70 | 0.11 | 112,098 |  |
| 57 |  | Feels part of the area | Strongly agree | 0 | 1.3 | 46.13 | 45.17 | 47.09 | 5,628,729 | 12,201,884 |
|  |  |  | Agree | 0.33 |  | 47.77 | 46.82 | 48.73 | 5,828,840 |  |
|  |  |  | Disagree | 0.66 |  | 5.26 | 4.87 | 5.68 | 642,063 |  |
|  |  |  | Strongly disagree | 1 |  | 0.83 | 0.70 | 1 | 101,764 |  |
| 58 |  | Vandalism | Strongly disagree | 0 | 1.3 | 41.57 | 40.63 | 42.51 | 5,101,394 | 12,271,816 |
|  |  |  | Disagree | 0.33 |  | 52.15 | 51.19 | 53.10 | 6,399,752 |  |
|  |  |  | Agree | 0.66 |  | 5.39 | 5 | 5.81 | 661,942 |  |
|  |  |  | Strongly agree | 1 |  | 0.89 | 0.75 | 1.07 | 109,808 |  |
| 59 |  | Feel lonely in the area | Strongly disagree | 0 | 0.9 | 36.39 | 35.48 | 37.31 | 4,462,998 | 12,264,352 |
|  |  |  | Disagree | 0.33 |  | 55.27 | 54.32 | 56.22 | 6,778,507 |  |
|  |  |  | Agree | 0.66 |  | 7.27 | 6.80 | 7.75 | 891,005 |  |
|  |  |  | Strongly agree | 1 |  | 1.07 | 0.90 | 1.26 | 131,106 |  |
| 60 |  | Most people trusted in the area | Strongly agree | 0 | 2.4 | 36.95 | 36.01 | 37.89 | 4,452,673 | 12,050,537 |
|  |  |  | Agree | 0.33 |  | 58.27 | 57.31 | 59.23 | 7,021,848 |  |
|  |  |  | Disagree | 0.66 |  | 4.04 | 3.65 | 4.47 | 487,203 |  |
|  |  |  | Strongly disagree | 1 |  | 0.74 | 0.60 | 0.92 | 89,078 |  |
| 61 |  | Afraid to walk in the area | Strongly disagree | 0 | 1.9 | 32.97 | 32.07 | 33.89 | 4,000,277 | 12,133,082 |
|  |  |  | Disagree | 0.33 |  | 55.71 | 54.75 | 56.66 | 6,759,340 |  |
|  |  |  | Agree | 0.66 |  | 9.75 | 9.20 | 10.33 | 1,182,975 |  |
|  |  |  | Strongly agree | 1 |  | 1.57 | 1.34 | 1.84 | 190,732 |  |
| 62 |  | Friendly people in the area | Strongly agree | 0 | 1.1 | 35.72 | 34.82 | 36.64 | 4,370,131 | 12,234,409 |
|  |  |  | Agree | 0.33 |  | 61.98 | 61.06 | 62.90 | 7,582,887 |  |
|  |  |  | Disagree | 0.66 |  | 2 | 1.74 | 2.29 | 244,199 |  |
|  |  |  | Strongly disagree | 1 |  | 0.30 | 0.22 | 0.41 | 36,459 |  |
| 63 |  | People take advantage in the area | Strongly disagree | 0 | 1.8 | 32.32 | 31.42 | 33.22 | 3,924,797 | 12,143,554 |
|  |  |  | Disagree | 0.33 |  | 62.92 | 61.99 | 63.85 | 7,640,724 |  |
|  |  |  | Agree | 0.66 |  | 4.21 | 3.80 | 4.65 | 510,879 |  |
|  |  |  | Strongly agree | 1 |  | 0.50 | 0.43 | 0.70 | 66,972 |  |
| 64 |  | Clean area | Strongly agree | 0 | 0.8 | 32.38 | 31.49 | 33.29 | 3,972,454 | 12,268,234 |
|  |  |  | Agree | 0.33 |  | 63.90 | 62.98 | 64.82 | 7,839,402 |  |
|  |  |  | Disagree | 0.66 |  | 3.28 | 2.96 | 3.63 | 401,907 |  |
|  |  |  | Strongly disagree | 1 |  | 0.40 | 0.33 | 0.58 | 54,054 |  |
| 65 |  | Help available in the area | Strongly agree | 0 | 3.2 | 33.43 | 32.53 | 34.35 | 3,999,244 | 11,963,039 |
|  |  |  | Agree | 0.33 |  | 61.54 | 60.60 | 62.48 | 7,362,054 |  |
|  |  |  | Disagree | 0.66 |  | 4.48 | 4.11 | 4.89 | 536,064 |  |
|  |  |  | Strongly disagree | 1 |  | 0.54 | 0.41 | 0.72 | 64,959 |  |
| 66 | Wealth | Savings | Yes | 0 | 0.1 | 98.76 | 98.44 | 99.02 | 12,207,254 | 12,360,525 |
|  |  |  | No | 1 |  | 1.24 | 0.98 | 1.56 | 153,023 |  |
| 67 |  | Life insurance | Yes | 0 | 0.9 | 75.78 | 74.97 | 76.57 | 9,280,233 | 12,246,283 |
|  |  |  | No | 1 |  | 24.22 | 23.43 | 25.03 | 2,966,050 |  |
| 68 |  | Assets | Yes | 0 | 0.9 | 91.71 | 91.12 | 92.26 | 11,335,837 | 12,360,525 |
|  |  |  | No | 1 |  | 8.29 | 7.74 | 8.88 | 1,025,058 |  |
| 69 |  | Debts | No | 0 | 0.1 | 52.51 | 51.56 | 53.46 | 6,490,512 | 12,360,525 |
|  |  |  | Yes | 1 |  | 47.49 | 46.54 | 48.44 | 5,870,013 |  |
| 70 |  | Self-rated financial status | Manage very well | 0 | 0.8  0.9 | 38.50 | 37.59 | 39.43 | 4,716,910 | 12,251,713 |
|  |  |  | Manage quite well | 0.2 |  | 34.39 | 33.50 | 35.30 | 4,213,364 |  |
|  |  |  | Get by alright | 0.4 |  | 22.13 | 21.29 | 22.98 | 2,711,304 |  |
|  |  |  | Don't manage very well | 0.6 |  | 1.25 | 1.07 | 1.46 | 152,779 |  |
|  |  |  | Have some financial difficulties | 0.8 |  | 3.20 | 2.87 | 3.57 | 392,300 |  |
|  |  |  | Have severe financial difficulties | 1 |  | 0.53 | 0.43 | 0.66 | 64,726 |  |
| 71 |  | Adequate income for basic needs | Very well | 0 |  | 48.15 | 47.20 | 49.11 | 5,897,777 | 12,248,759 |
|  |  |  | Adequately | 0.25 |  | 40.83 | 39.88 | 41.79 | 5,001,168 |  |
|  |  |  | With some difficulty | 0.5 |  | 8.36 | 7.83 | 8.93 | 1,024,486 |  |
|  |  |  | Not very well | 0.75 |  | 1.75 | 1.53 | 1.99 | 213,863 |  |
|  |  |  | Totally inadequately | 1 |  | 0.90 | 0.75 | 1.10 | 111,194 |  |
| 72 |  | Little money stops from doing things | No | 0 | 0.1 | 64.72 | 63.79 | 65.64 | 7,999,732 | 12,360,525 |
|  |  |  | Yes | 1 |  | 35.28 | 34.36 | 36.21 | 4,360,793 |  |
| 73 |  | Insufficient financial resources in the future | Little or no possibility | 0 | 2.5 | 61.27 | 60.33 | 62.21 | 7,383,815 | 12,051,273 |
|  |  |  | Some possibility | 0.5 |  | 33.06 | 32.16 | 33.98 | 3,984,151 |  |
|  |  |  | High possibility | 1 |  | 5.66 | 5.23 | 6.13 | 682,584 |  |
| 74 |  | Leave inheritance | High | 0 | 3.8 | 41.77 | 40.82 | 42.72 | 4,969,184 | 11,896,537 |
|  |  |  | Low | 0.33 |  | 24.71 | 23.85 | 25.58 | 2,939,634 |  |
|  |  |  | Moderate | 0.66 |  | 12.84 | 12.17 | 13.54 | 1,527,515 |  |
|  |  |  | None | 1 |  | 20.69 | 19.91 | 21.49 | 2,461,394 |  |
